# Coronary Artery Disease in Women: Sex-Specific Pathophysiology, Risk Factors, Clinical Presentation and Management

**DOI:** 10.3390/medicina62071313

**Published:** 2026-07-07

**Authors:** Kassiani-Maria Nastouli, Anastasios Apostolos, Maria Bozika, Georgios Boliaris, Panagiotis Iliakis, Nikolaos Ktenopoulos, Panayotis K. Vlachakis, Paschalis Karakasis, Theoni Theodoropoulou, Nikolaos Tsiamis, Nikias Milaras, Anna Pitsillidi, Konstantinos Konstantinou, Ioannis Skalidis, Konstantinos Toutouzas, Konstantinos Tsioufis, Vasileios Panoulas

**Affiliations:** 1 Department of Medicine, Division of Cardiology, University Hospital of Patras, 26504 Patras, Greece; kassienmarie@gmail.com (K.-M.N.); mariabozika29@gmail.com (M.B.); gmpoliaris@gmail.com (G.B.); 2 Department of Cardiology, Royal Brompton and Harefield Hospitals, Guy’s and St Thomas’ NHS Foundation Trust, London UB9 6JH, UK; vasileios.panoulas2@nhs.net; 3 Faculty of Medicine, Imperial College London, London SW7 2AZ, UK; 4 Department of Medicine, Division of Cardiology, Angiology and Internal Emergency Medicine, Ruhr University Bochum, Knappschaft Kliniken University Hospital Bochum, 44892 Bochum, Germany; panayiotisiliakis@gmail.com; 5 First Cardiology Department, School of Medicine, Hippokration General Hospital, National and Kapodistrian University of Athens, 11527 Athens, Greece; nikosktenop@gmail.com (N.K.); vlachakispanag@gmail.com (P.K.V.); theoni.theodoropoulou98@gmail.com (T.T.); nik.tsiamis@gmail.com (N.T.); kostiskon@gmail.com (K.K.); ktoutouz@gmail.com (K.T.); ktsioufis@gmail.com (K.T.); 6 Second Department of Cardiology, Hippokration General Hospital, Aristotle University of Thessaloniki, 54124 Thessaloniki, Greece; pakar15@hotmail.com; 7 Cardiology Department, Sismanogleio General Hospital, 15126 Athens, Greece; 8 Department of Cardiology, “Hippokration” General Hospital, 11527 Athens, Greece; nikiasmilaras@gmail.com; 9 Department of Obstetrics and Gynaecology, Rheinland Klinikum Neuss, Preußenstrasse 84, 41464 Neuss, Germany; anna.pitsillidi@gmail.com; 10 Department of Cardiology, University and Hospital Fribourg, 1708 Fribourg, Switzerland; skalidis7@gmail.com; 11 National Heart and Lung Institute, Imperial College London, London SW7 2AZ, UK

**Keywords:** coronary syndromes, women, sex differences, INOCA, MINOCA, spontaneous coronary artery dissection, coronary microvascular dysfunction, cardiovascular risk

## Abstract

Cardiovascular disease remains the leading cause of mortality among women worldwide, yet coronary syndromes in women continue to be under-recognized and insufficiently represented in clinical research. This review summarizes sex-specific pathophysiological mechanisms, risk factors, clinical presentation, and management considerations in women with coronary syndromes. Women are more likely than men to present with non-obstructive and non-atherosclerotic ischemic phenotypes, including ischemia or angina with non-obstructive coronary arteries, coronary microvascular dysfunction, myocardial infarction with non-obstructive coronary arteries, spontaneous coronary artery dissection, vasospastic angina, and Takotsubo syndrome. These entities often require diagnostic strategies beyond the detection of flow-limiting epicardial stenosis, including cardiac magnetic resonance imaging, intracoronary imaging, and coronary function testing. Traditional cardiovascular risk factors remain important, but several female-specific risk enhancers, including premature menopause, adverse pregnancy outcomes, polycystic ovary syndrome, autoimmune disease, and psychosocial stressors, further modify risk and remain incompletely integrated into routine clinical assessment. Women may also experience diagnostic delays due to symptom misclassification, lower baseline troponin concentrations, and clinical algorithms historically derived from male-predominant populations. Management should follow guideline-directed therapy when appropriate, while recognizing sex-related differences in pharmacology, bleeding risk, revascularization outcomes, and the need for phenotype-specific treatment in INOCA, MINOCA, SCAD, and Takotsubo syndrome. Finally, transgender and gender-diverse individuals remain largely absent from cardiovascular trials, highlighting the need for inclusive research frameworks that distinguish sex, gender identity, and hormone exposure. Improved recognition of sex- and gender-related differences is essential to advance equitable cardiovascular care.

## 1. Introduction

Cardiovascular disease (CVD) remains the leading cause of mortality in men and women worldwide, with ischemic heart disease (IHD) and stroke accounting for most female CVD fatalities [1]. Yet, despite this enormous impact, women’s cardiovascular health has long been neglected, and a substantial proportion of affected women remain underdiagnosed while data mainly depend on male study populations [2,3]. Part of the issue is that coronary artery disease (CAD) in women differs from the traditional obstructive epicardial atherosclerosis frequently observed in males. Although women develop conventional atherosclerotic CAD, they are more likely than men to experience diffuse atherosclerosis, ischemia with non-obstructive coronary arteries (INOCA), angina with non-obstructive coronary arteries (ANOCA), coronary microvascular dysfunction (CMD), vasomotor disorders, myocardial infarction with non-obstructive coronary arteries (MINOCA), spontaneous coronary artery dissection (SCAD), and stress cardiomyopathy [4]. These pathophysiological differences contribute to diagnostic uncertainty, especially when clinical algorithms and diagnostic thresholds prioritize the detection of flow-limiting epicardial stenosis [5]. As a result, women with ischemic symptoms may be falsely reassured after non-obstructive angiographic findings, despite ongoing ischemia, impaired quality of life, and increased cardiovascular risk.

Sex-specific and gender-related differences further shape the clinical presentation, risk profile, and management of CAD. Women more frequently present with symptoms that are labelled “atypical,” although this terminology may reflect the use of male-pattern symptom models rather than true atypicality [6]. In parallel, traditional risk factors may exert different relative effects in women, while female-specific risk enhancers, remain incompletely integrated into routine cardiovascular risk assessment and scarcely examined in clinical trials [7].

Constant under-representation of women in cardiovascular trials has confined the ability to define disease mechanisms, treatment responses, drug safety, bleeding risk, and potential outcomes specifically targeted in women [8]. This absence in information becomes substantially more complicated when considering transgender and gender-diverse individuals, whose participation and report is absent from cardiovascular trials. The American Heart Association has highlighted that data on cardiovascular health in transgender and gender-diverse populations remain limited, while current evidence suggests that several environmental, societal factors and barriers to health care may all influence the total cardiovascular risk [9]. A recent analysis further reported that these populations are not reported in almost all contemporary cardiovascular clinical trials, leading to a missing representation in cardiovascular research [10]. 

This review, based on the Sex and Gender Equity in Research (SAGER) guidelines, distinguishes between sex and gender as related but distinct constructs. Sex refers to biological attributes, including chromosomal, anatomical, hormonal, and physiological characteristics, whereas gender refers to identity, roles, behaviors, and sociocultural experiences that may influence cardiovascular risk, healthcare access, and clinical outcomes [11]. Throughout this review, the term “women” is used primarily to reflect the populations included in most cardiovascular studies cited, which have generally relied on sex assigned at birth or binary sex categories. Transgender and gender diverse individuals are addressed separately, given that report on gender identity, hormone exposure, and gonadal status currently remains absent from cardiovascular research.

The aim of this review is to summarize the sex-specific pathophysiological mechanisms, risk factors, clinical presentation, and management of CAD in women. In addition, it briefly highlights the limited evidence regarding transgender and gender-diverse individuals as an important area for future cardiovascular research and trial inclusion.

## 2. Methods

The present review was conducted as a narrative literature review. A targeted search of PubMed/MEDLINE, Scopus, Web of Science, and Google Scholar was performed to identify relevant publications on sex- and gender-related differences in coronary artery disease. The literature search covered all available records from database inception to May 2026, with the final search performed in May 2026.

The search strategy used combinations of terms related to coronary artery disease, ischemic heart disease, acute coronary syndrome, women, sex differences, gender differences, INOCA, MINOCA, coronary microvascular dysfunction, spontaneous coronary artery dissection, Takotsubo syndrome, menopause, pregnancy-related complications, transgender and gender-diverse populations, and cardiovascular clinical trials. Reference lists of relevant original articles, reviews, scientific statements, consensus documents, position papers, and clinical guidelines were also manually screened.

Eligible publications included original studies, randomized clinical trials, systematic reviews, meta-analyses, narrative reviews, scientific statements, consensus documents, position papers, clinical guidelines, reports, and relevant book chapters published in English. Studies were considered eligible if they addressed sex- or gender-specific aspects of coronary artery disease, female-predominant coronary syndromes, reproductive or hormonal cardiovascular risk factors, women’s representation in cardiovascular clinical trials, or cardiovascular considerations in transgender and gender-diverse populations.

Exclusion criteria included non-English publications, duplicate articles, conference abstracts without sufficient methodological or clinical detail, articles outside the scope of the review, and studies without relevant sex- or gender-specific information. Evidence was selected according to clinical relevance and synthesized thematically. Because this was conducted as a narrative literature review, no formal risk-of-bias assessment, study quality scoring, or quantitative synthesis was performed.

## 3. Sex-Specific Pathophysiology

Acute coronary syndromes include a spectrum of clinical presentations caused by acute myocardial ischemia, most related to plaque disruption and thrombosis, but also including non-obstructive and non-atherosclerotic mechanisms that are particularly frequent in women. However, acute ischemic presentations more frequently include non-obstructive or non-atherosclerotic mechanisms, such as MINOCA, CMD, SCAD and Takotsubo cardiomyopathy (Table 1) [12]. Although broader conceptual terms have been proposed to capture these entities, “acute coronary syndrome” remains the standard terminology used in major clinical guidelines.

As we tackle the reasons behind sex-specific differences in ACS and generally CAD causes, oestrogen appears to be the primary candidate for explaining sex-related differences. The pathophysiology of atherosclerotic ACS in women illustrates the varied and sometimes opposing effects of oestrogen. Plaque rupture generally involves disruption of a thin fibrous cap with exposure of the thrombogenic necrotic core, whereas plaque erosion is characterized by a thrombus formation over an intact or superficially injured plaque surface [13]. Erosion is the most common cause of ACS in women under 50 years old (73%), with rupture accounting for 26% of the cases. This ratio however changes with age, as rupture incidence increases and represents 70% of ACS in women above the age of 80 [14]. Estrogen and other sex hormones likely contribute to vascular homeostasis through effects on endothelial function, nitric oxide bioavailability, lipid metabolism, inflammatory pathways, and vascular tone. Experimental and clinical evidence suggests that estrogen may exert vasodilatory, anti-inflammatory, and anti-atherogenic effects through modulation of the renin–angiotensin system, enhancement of nitric oxide production, favorable lipid regulation, and attenuation of oxidative stress. Its decline after menopause may therefore contribute to endothelial dysfunction, hypertension, adverse lipid profile changes, and increased cardiovascular risk. However, these mechanisms interact with ageing, smoking, diabetes, obesity, and other cardiometabolic factors; thus, estrogen deficiency should not be viewed as the sole explanation for sex-specific differences in ACS and CAD [14]. Women presenting with ACS were more likely to have diabetes than their male counterparts [14,15]; a paradoxical finding considering the antidiabetic estrogen effect. Therefore, it may be deduced that diabetes presents a risk factor more aggressive in women, overcoming the estrogenic protecting effect. Lastly, it is worth mentioning that plaque erosion to rupture ratio is reversed in female smokers. Smoking is known to decrease estrogen levels, thus enhancing evidence of estrogen in atheromatic ACS protection [14].

Although estrogen-related vascular effects are important, sex-specific cardiovascular disease cannot be explained by hormonal mechanisms alone. Genetic and epigenetic factors, including sex-chromosome effects and sex-biased gene expression, may influence vascular biology, endothelial function, inflammation, and myocardial remodelling [16,17]. Inflammatory and autoimmune disorders, which are more prevalent in women, may further promote endothelial dysfunction and coronary microvascular disease, while metabolic risk factors such as diabetes, obesity, and dyslipidaemia may confer different relative cardiovascular risks in women and men [18]. Psychosocial factors, including depression, chronic stress, socioeconomic disadvantage, and gender-related differences in healthcare access, may also modify symptom perception, diagnostic delay, treatment delivery, and outcomes. Therefore, sex-specific cardiovascular pathophysiology should be viewed as the result of interacting hormonal, genetic, inflammatory, metabolic, and psychosocial determinants rather than estrogen deficiency alone.

Non-obstructive and non-atherosclerotic mechanisms consist a major influence in ischemic syndromes among women. SCAD, which predominantly affects women, is characterized by separation of the coronary arterial wall and formation of an intramural hematoma leading to compression of the true lumen and impaired coronary blood flow [19]. Although the exact mechanisms remain incompletely characterized, proposed contributors include arteriopathies, hormonal and hemodynamic influences, pregnancy-related vascular changes, connective tissue vulnerability, virus infections and vascular tortuosity. The increased incidence of SCAD during the peripartum period further supports a potential role of hormonal and vascular remodelling factors [20,21]. Additionally, thyroid dysfunction, especially autoimmune thyroid disease and hypothyroidism, is more common in women and has been suggested as a potential predisposing or prognostic factor in SCAD. Recent registry data indicate that SCAD patients with hypothyroidism may display a more diffuse angiographic phenotype and higher recurrence/adverse event rates, although causality remains unproven [22]. Another important factor that needs to be addressed is SCAD results as a combination of both a predisposing vascular substrate and acute precipitating triggers [23,24]. Predisposing factors include female sex, pregnancy/peripartum-related hormonal and haemodynamic changes, fibromuscular dysplasia or other extracoronary arteriopathies, connective tissue disorders, systemic inflammatory disease, migraine, thyroid disease, and genetic susceptibility [25]. Precipitating factors, in contrast, are acute stressors that may increase coronary shear stress, including emotional stress, intense physical exertion, and Valsalva-like manoeuvres such as labour pushing, heavy lifting, coughing, retching/vomiting, defecation, or sexual activity. In the peripartum setting, Valsalva during labour should therefore be considered a potential trigger superimposed on pregnancy-related vascular vulnerability, rather than a sole causal mechanism, especially because many pregnancy-associated SCAD events occur postpartum rather than during delivery [26]. Sex-specificity should also be clearly delineated: pregnancy-related and hormonal factors are specific to women, emotional stress is more frequently reported in women, whereas physical exertion and Valsalva-type triggers appear relatively more common among men.

MINOCA is defined as an entity, characterized by the presence of acute myocardial infarction criteria, absence obstructive coronary stenosis ≥50% in any major epicardial vessel and no clinically detected alternative explanation at the time of angiography, requiring further investigation for certain diagnosis [27]. Women are disproportionately affected, comprising nearly 50% of the MINOCA population but only 25% of obstructive AMI cases, and are more than twice as likely as men to present this way. Its mechanistic breadth is considerable: plaque disruption is identified on intracoronary imaging in approximately one third of cases likely an underestimate given that erosion requires OCT rather than IVUS while coronary vasospasm is detected in up to 46% of patients undergoing provocative testing [27,28]. Microvascular dysfunction, present in 30–50% of those with non-obstructive coronaries and chest pain, may cause ischemia directly or be an outcome of myocardial injury. Cardiac Magnetic Resonance Imaging (CMR) is central to the workup, identifying an underlying cause in up to approximately 74% of cases, including myocarditis present in 33% of patients fulfilling MINOCA criteria [29]. Taking into account the heterogeneity in presentation, a common treatment strategy among patients is not suitable and management should directly target the respective mechanism.

ANOCA/INOCA should be considered as a heterogenous spectrum of coronary vascular disorders, including several phenotypes with CMD with impaired vasodilatory reserve, microvascular angina, epicardial vasospastic angina, microvascular spasm, mixed vasomotor dysfunction, diffuse non-obstructive atherosclerosis, myocardial bridging, and, in selected patients, non-cardiac chest pain after normal coronary functional assessment. This distinction is relevant in women, who are less represented among patients referred for angina or objective ischaemia without obstructive epicardial CAD; estimates suggest that INOCA is identified in approximately 50–70% of women compared with 30–50% of men undergoing coronary angiography for angina [30].

Endotypes of ANOCA/INOCA has been indicated to differ by sex. Specifically, Jansen et al. in a perspective cohort of patients with angina and non-obstructive coronary arteries undergoing comprehensive invasive coronary function testing with acetylcholine and adenosine, found that coronary vasomotor dysfunction was highly prevalent in both sexes, but with different patterns: men more frequently demonstrated epicardial spasm, whereas women more often showed microvascular spasm and impaired coronary flow reserve [31]. Specifically, epicardial spasm was observed in 63% of men versus 42% of women, while microvascular spasm was observed in 29% of men versus 40% of women; impaired coronary flow reserve was also more frequent among women.

These findings support the concept that women with angina and non-obstructive coronary arteries are more likely to have microvascular involvement, either through impaired vasodilatory capacity, microvascular spasm, or mixed microvascular-vasospastic disease. This is clinically important because conventional angiography alone may exclude flow-limiting epicardial stenosis but cannot reliably diagnose coronary microvascular dysfunction or vasospasm. Invasive coronary function testing, including acetylcholine provocation and adenosine-based assessment of coronary flow reserve and microvascular resistance, can therefore help identify the dominant endotype and guide mechanism-based therapy [32].

CMD may exist in the absence of cardiovascular disease, with cardiomyopathies or obstructive CAD, or be iatrogenic by distal emboli. Reactive Oxygen Species (ROS) affecting the healthy balance between vasodilatory Nitric Oxide (NO) and constricting endothelin are key molecular mechanisms associated with endothelial cell (EC) function. CMD can additionally be endothelium-independent, when smooth muscle cell responses to vasodilatory or constricting signals are abnormal. Adverse arteriole remodeling is another contributing factor [33].

Vasospastic angina follows a similar but distinct pattern and is more commonly found in women. Teragawa et al. found that Nitroglycerin induced dilation (NID) of the brachial artery was higher in women under 60 years old diagnosed with vasospastic angina, while flow-mediated dilation (FMD) did not differ compared to women over 60 [33]. A potential explanation for this observation is that endothelial dysfunction and/or smooth muscle cells hypercontraction, are possibly influenced by the decline in sex hormones post-menopause, although additional factors like smoking prevalence among age group confound this assumption [34].

Takotsubo cardiomyopathy represents a traditionally non-coronary acute cardiac pathology (a statement recently challenged by the literature), where a catecholamine-mediated adrenergic surge causes transient ventricular dysfunction, famously resulting in the characteristic apical “ballooning” pattern [35]. Its ninefold predominance in women likely reflects higher sympathetic nervous activity and the negative emotional stress that may induce it, both factors more commonly found in female patients [19].

## 4. Risk Factors for CAD

### 4.1. Traditional Risk Factors

Traditional cardiovascular risk factors remain central to the development of atherosclerotic disease between women and men. These include modifiable factors, such as hypertension, diabetes mellitus, dyslipidemia, obesity, smoking, physical inactivity, and psychosocial stress, as well as non-modifiable factors, including age, ethnicity, and family history of premature CAD [36,37,38]. The risk of developing CAD increases with age, with men presenting a 49% lifetime risk, while women have a 32% risk of developing symptomatic CAD [39]. Family history remains an important marker of inherited and shared environmental susceptibility to premature CAD [odds ratio (OR): 9.0, 95% CI: 4.7–17.3] [40]. In FRATER study 480 patients underwent coronary computed tomography angiography (CCTA). Those with a family history of CAD had a significantly higher prevalence of high risk atherosclerosis than matched controls without family history (62% vs. 43%, *p* = 0.0001) [41]. Interestingly, even among patients classified as having very-low clinical probability of obstructive CAD, high-risk atherosclerosis or obstructive CAD was detected in 42% of those with a family history compared with 19% of those without, supporting the added value of family history in cardiovascular risk assessment. A family history of premature atherosclerotic cardiovascular disease should prompt earlier and more intensive risk assessment, particularly when combined with female-specific risk enhancers [42]. Ethnicity and social determinants of health can also influence CAD burden, with higher cardiovascular morbidity and mortality reported among Black, Hispanic/Latina, and South Asian populations [43,44,45].

Hypertension is an important modifiable risk factor for CAD and cardiovascular morbidity among women [46]. Its impact increases after midlife, partly due to vascular aging, menopause-associated endothelial dysfunction, activation of neurohormonal pathways, and increasing arterial stiffness [47]. Cardiovascular risk in women may rise from blood pressure levels around 130/80 mmHg, suggesting that conventional thresholds may underestimate risk in some women [48]. Hypertension is also strongly linked to target-organ damage, including left ventricular hypertrophy and heart failure with preserved ejection fraction, particularly when accompanied by obesity, renal dysfunction, or metabolic disease [49].

Diabetes mellitus is another major risk factor with particular importance in women. The disease was associated with an approximately 44% higher risk of fatal coronary heart disease in women than in men, independently of other established cardiovascular risk factors [50]. Potential mechanisms include greater endothelial dysfunction, more pronounced systemic inflammation, unfavorable changes in coagulation and fibrinolysis, and a tendency toward more diffuse and microvascular coronary disease in diabetic women. Thus, diabetes should be regarded as a powerful CAD risk enhancer in women and warrants aggressive glycaemic control alongside comprehensive cardiovascular risk management, alongside comprehensive cardiovascular risk factor management [51].

Dyslipidemia remains a fundamental driver of atherosclerosis in women and statin therapy remains central to primary and secondary prevention. A large-scale meta-analysis confirmed that relative reduction in the major vascular events with statin therapy was comparable between women and men (*p* = 0.33), supporting efficacy regardless of sex [52]. Moreover, lipid patterns vary across reproductive stages. LDL cholesterol and triglycerides rise normally during pregnancy, while declining estrogen after menopause is associated with increases in LDL and non-HDL cholesterol [53,54,55]. Despite this, women with established or high-risk cardiovascular disease are less likely to receive guideline-recommended lipid-lowering therapy, as was suggested by the results of a study conducted by Nanna et al. (36.7% versus 45.2%; *p* < 0.001), with physicians less likely to prescribe statins in women in comparison to men (67.0% versus 78.4%, *p* < 0.001), further expanding the gap in treatment [56].

Obesity and physical inactivity are independent risk factors in women, and also promote hypertension, insulin resistance, dyslipidemia, inflammation, and heart failure with preserved ejection fraction [51]. Data from the Framingham Heart Study demonstrated that overweight and obesity independently increase cardiovascular risk in both sexes, with a graded rise across body mass index categories [57]. In women, menopause-related visceral adiposity, systemic inflammation, and vascular ageing further amplify the risk [58].

Several sex-specific conditions substantially modify cardiovascular risk in women and are currently re important determinants of CAD.

### 4.2. Menopause and Premature Menopause

Menopause is associated with a significant change in cardiovascular risk profile. It is associated with adverse cardiometabolic changes, including elevations in total and LDL cholesterol, reductions in HDL cholesterol, increases in body weight and central adiposity, fasting glucose and insulin levels [58,59]. Longitudinal data from the Study of Women’s Health Across the Nation (SWAN) suggest that these changes accelerate during the menopausal transition itself, rather than reflecting ageing alone, supporting a direct hormonal contribution to midlife cardiovascular risk [60].

Premature menopause, defined as occurring before the age of 40, and early menopause, occurring between ages 40 and 44, are each independently associated with increased cardiovascular risk. In a pooled analysis of over 203,767 patients, a relationship between age at natural menopause and cardiovascular event rates was found, with women experiencing premature surgical menopause facing the highest risk (HR: 1.22, 95% CI 1.16–1.28) [61]. Honigberg and colleagues, using data from a large biobank cohort, demonstrated that premature natural menopause and premature surgical menopause were independently associated with increased cardiovascular risk [(HR: 1.36 (95% CI, 1.19–1.56; *p* < 0.001) for natural menopause and 1.87 (95% CI, 1.36–2.58; *p* < 0.001) for surgical menopause] [62]. However, whether premature menopause is a causal or a marker of pre-existing vascular vulnerability remains incompletely understood.

### 4.3. Pregnancy-Related Disorders

Pregnancy-related complications have no direct equivalent in men and are associated with long-term cardiovascular risk. Adverse pregnancy outcomes, including preeclampsia, gestational diabetes, and spontaneous preterm delivery, are independently associated with cardiovascular morbidity [63].

Preeclampsia complicates roughly 3–5% of pregnancies and is associated with higher risk of hypertension (HR: 4.47; 4.32–4.62), IHD (HR: 1.67; 1.54–1.81), heart failure (HR: 2.13; 1.64–2.76), cerebrovascular disease (HR: 1.9; 1.53–2.35), independent of pre-pregnancy medical history [64]. Gestational diabetes similarly is linked with a twofold increase in cardiovascular events compared to patients with normal glycemic blood levels [Risk Ratio (RR) 1.98, 95% Confidence Interval (CI) 1.57–2.50], reflecting an underlying metabolic vulnerability [65]. Similarly, spontaneous preterm delivery has been linked to increased cardiovascular risk, potentially through shared pathways of vascular inflammation, abnormalities in coagulation and placental insufficiency [66].

### 4.4. Polycystic Ovary Syndrome (PCOS)

Polycystic ovary syndrome (PCOS) recently renamed as polyendocrine metabolic ovarian syndrome (PMOS) is linked with an altered cardiometabolic profile and affects approximately 5–13% of women of reproductive age [67,68]. It is characterized by hyperandrogenism, ovulatory dysfunction and polycystic ovarian morphology. Women with PCOS have higher rates of insulin resistance, obesity, metabolic syndrome, dyslipidemia, and hypertension, which all represent established contributors to CAD [69,70,71].

A meta-analysis by Zhao and colleagues demonstrated that patients with PCOS have increased risk of CHD (OR = 1.30, 95% CI 1.09–1.56; *p* = 0.004) [72]. Furthermore, Zhou and colleagues reported that PCOS is associated with an elevated risk of stroke (OR = 1.36, 95% CI 1.09–1.70; *p* = 0.007) [73]. The excess cardiovascular risk likely involves the combined effects of insulin resistance, hyperandrogenism, endothelial dysfunction, and pro-inflammatory and pro-thrombotic states that characterize the condition.

### 4.5. Systemic Inflammatory and Autoimmune Disorders

Systemic inflammatory and autoimmune disorders e.g., rheumatoid arthritis, systemic lupus erythematosus, disproportionally affect women and confer cardiovascular risk through the process of chronic inflammation, immune-mediated vascular injury, corticosteroid use, through pathways which are not captured by the traditional scores [74,75].

### 4.6. Psychosocial Risk Factors

A significant risk factor for the provocation of CAD that is frequently overlooked when women’s health is evaluated, is physical and psychological abuse by intimate partners, experienced by a substantial proportion of women throughout their lifetime [76]. Chronic stress from abuse and depression directly affect cardiovascular health, whereas violence may also affect ubiquitous health behavior, with increasing substance abuse and self-neglect [77]. Lower HDL (*p* = 0.031), and elevated triglycerides (*p* = 0.003) were observed with increased abdominal obesity (*p* = 0.001) [78].

Interestingly, a connection between depression and SCAD has been observed. In a Canadian study depression was reported more frequently in women than in men with SCAD (20.2% vs. 9.8%; *p* = 0.005), alongside a higher prevalence of emotional stress triggers (59.3% vs. 35.0%; *p* < 0.001) and high perceived stress scores (11.0% vs. 3.5%; *p* = 0.025) [79,80]. However, whether depression represents a true predisposing factor, a marker of sex-related vulnerability, or a bystander reflecting the higher background prevalence of depression among women remains uncertain.

## 5. Clinical Presentation

The clinical manifestations of coronary disease also differ. Chest pain may constitute the most frequent clinical presentation, though women are more likely to present without chest pain, reporting symptoms like nausea and dizziness, dyspnea, fatigue, jaw pain, palpitations and vomiting, usually characterized as atypical [81]. These so-called “atypical” presentations are, in fact, the most common presentation of the disease among women and their misclassification as atypical contributes to diagnostic delays and missed diagnoses [1,81].

Sex-specific differences are also important in the evaluation of chronic coronary syndromes. Recent guidelines emphasize individualized clinical likelihood assessment and include an expanded framework for angina or ischemia with no or non-obstructive coronary arteries, now termed ANOCA/INOCA [82]. This is particularly relevant in women, who more frequently have non-obstructive coronary disease, coronary microvascular dysfunction, or vasomotor disorders despite persistent ischemic symptoms. Therefore, the absence of flow-limiting epicardial stenosis should not be interpreted as absence of ischemic heart disease when symptoms persist [82]. Contemporary diagnostic approaches support endotype-based evaluation, including coronary flow reserve, microvascular resistance assessment, and acetylcholine provocation to identify impaired vasodilatory capacity, epicardial vasospasm, microvascular spasm, or coronary microvascular dysfunction, thereby enabling more targeted antianginal and vasodilator therapy [83].

Clinical prediction models and pre-test probability tools have historically demonstrated lower diagnostic performance in women, partly because women have lower rates of obstructive epicardial CAD and higher rates of ANOCA/INOCA. Non-invasive testing should therefore be selected according to clinical likelihood, symptom profile, risk factors, and the suspected mechanism of ischemia. Coronary CT angiography is useful for excluding obstructive CAD and characterizing plaque, whereas functional stress imaging may help identify ischemia [84,85]; however, tests focused only on epicardial obstruction may miss microvascular or vasospastic disease [30,82]. Furthermore, Coronary CT angiography enables detailed plaque phenotyping, permitting identification of high-risk features including low-attenuation non-calcified plaque, positive arterial remodelling, spotty calcification, and the napkin-ring sign [86,87]. Concurrently, the pericoronary fat attenuation index offers a non-invasive surrogate of localized coronary inflammation [88]. These modalities may prove especially valuable in female patients, who frequently present with anginal symptoms and myocardial ischaemia in the absence of obstructive epicardial stenoses, and in whom calcium-based scoring may underestimate risk attributable to non-calcified or inflamed atherosclerotic burden. Accordingly, integrating CCTA-derived plaque characterization with pericoronary inflammation assessment could augment traditional calcium scoring and facilitate more precise individualized risk evaluation. Nonetheless, prospective sex-stratified studies remain necessary to validate the incremental prognostic utility of these imaging biomarkers across diverse populations.

Another important factor is that women are more often older at presentation, and more frequently have comorbidities such as hypertension, diabetes, chronic kidney disease, or heart failure. In addition, women have a higher relative burden of MINOCA, SCAD, coronary vasospasm, coronary microvascular dysfunction, and Takotsubo syndrome, which should be considered in women presenting with acute chest pain, ischemic electrocardiographic changes, troponin elevation, or acute myocardial injury without obstructive coronary disease. In suspected MINOCA, a simplified diagnostic algorithm should begin with confirmation of myocardial infarction with non-obstructive coronary arteries and exclusion of overt non-cardiac causes [89]. Early CMR can help distinguish true ischaemic MINOCA from mimics such as myocarditis or stress cardiomyopathy, while OCT/IVUS may identify plaque disruption, thrombus, intramural haematoma, or subtle SCAD, and coronary function testing can diagnose epicardial spasm, microvascular spasm, or microvascular dysfunction. This stepwise approach supports mechanism-based diagnosis and tailored therapy.

Moreover, interpretation of high-sensitivity cardiac troponin results in women requires caution. Indeed, women generally have lower troponin concentrations than men, and uniform diagnostic thresholds may contribute to under-recognition of myocardial infarction in some women. In the High-STEACS trial, implementation of sex-specific hs-cTnI thresholds increased the detection of myocardial injury by 42% in women compared with 6% in men, using assay-specific cut-offs of 16 ng/L for women and 34 ng/L for men. However, improved biomarker sensitivity alone did not eliminate downstream disparities in treatment or improve outcomes, underscoring that sex-specific thresholds must be integrated with serial troponin changes, clinical presentation, ECG findings, imaging, and equitable evidence-based management [19,90].

## 6. Management

### 6.1. Obstructive CAD

The management of CAD in women broadly adheres to the guideline-directed therapy established in general populations, including agents, lipid-lowering agents, beta-blockers, and renin-angiotensin-aldosterone system inhibitors when indicated [82]. However, sex-related pharmacokinetic and pharmacodynamic differences may influence drug responses [91,92]. In general, women have lower body mass index with higher body fat percentage, lower glomerular filtration rates, and different hepatic enzyme expression profiles, factors that may affect drug metabolism and clearance [93]. These differences are particularly relevant for antiplatelet therapy, where women may exhibit higher residual platelet reactivity on standard doses of clopidogrel and may be more susceptible to bleeding complications with potent antiplatelet agents [19,94].

Statins are comparably efficacious in women and men for reducing major vascular events; yet women eligible for statin therapy remain less likely than men to receive statin prescription in clinical practice (67% vs. 78.4%; *p* < 0.001) or guideline-recommended statin intensity (36.7% vs. 45.2%; *p* < 0.001) [52,56].

In the EMMY trial subanalysis, women represented only 17.7% of participants (84/476), while compared with men, women were older at presentation [61 (56–65) vs. 56 (51–64) years; *p* = 0.005] and had higher baseline NT-proBNP levels [2117 (1383–3267) vs. 1137 (695–2050) pg/mL; *p* < 0.001] [91]. However, the beneficial effects of early empagliflozin treatment after AMI were not modified by sex, including effects on NT-proBNP (P_interaction_ = 0.984) and left ventricular ejection fraction (P_interaction_ = 0.812).

Regarding revascularization, women undergoing percutaneous coronary intervention (PCI) or coronary artery bypass grafting (CABG) often present at older age, with a higher burden of comorbidities, contributing to greater procedural complexity and worse outcomes post procedure [95]. In ACS, sex-related treatment gaps persist: women with ST-elevation myocardial infarction (STEMI) have a 7% lower probability of undergoing angiography and a 21% lower probability of receiving revascularization, while women with ACS have a 17% lower probability of receiving non-aspirin antiplatelet therapy and more than two-fold higher rates of in-hospital major bleeding compared with men [96]. Anatomical factors, including smaller epicardial coronary arteries, may further increase procedural complexity [97]. These data support individualized revascularization decisions, radial-preferred access when feasible, careful device selection, and weight-, renal-, and age-adjusted antithrombotic dosing [1,82]. In a registry of 23,473 ACS patients undergoing cardiac catheterisation during index hospitalisation, coronary revascularisation was less frequent in women than in men (51.8% vs. 66.1%); women also had higher rates of bleeding or transfusion (12.8% vs. 7.3%) [98].

Importantly, women often have smaller coronary vessel calibre, greater coronary tortuosity, and a higher burden of microvascular dysfunction, which may increase procedural complexity, influence coronary flow, and contribute to worse outcomes despite contemporary reperfusion strategies. In the ISACS-STEMI-COVID-19 registry, the pandemic was associated with a similar reduction in primary PCI procedures in both sexes, but 30-day mortality increased significantly among women compared with the pre-pandemic period (12.1% vs. 8.7%; adjusted HR 1.66, 95% CI 1.31–2.11; *p* < 0.001), whereas this increase was not significant in men (5.8% vs. 6.7%; adjusted HR 1.14, 95% [24] CI 0.96–1.34; *p* = 0.12) [99]. More recent ISACS-STEMI-COVID-19 data further showed persistent sex disparities among STEMI patients undergoing mechanical reperfusion: women had higher in-hospital mortality (8.3% vs. 5.0%; adjusted HR 1.26, 95% CI 1.06–1.50; *p* = 0.01) and higher 30-day mortality (10.3% vs. 6.2%; adjusted HR 1.22, 95% CI 1.06–1.38; *p* = 0.007) [100]. These findings support the need to consider sex-specific coronary anatomy, vascular physiology, reperfusion delays, and procedural factors when interpreting outcomes in obstructive CAD.

### 6.2. Menopausal Hormone Therapy and Cardiovascular Risk

The cardiovascular implications of menopausal hormone therapy (MHT) represent one of the most debated and clinically consequential topics in women’s cardiovascular health. Early observational data from the Nurses’ Health Study suggested a cardioprotective effect of estrogen, generating widespread enthusiasm for MHT as a primary prevention strategy [101,102]. However, the Women’s Health Initiative (WHI) randomised trials, which enrolled postmenopausal women with a mean age of 63 years, reported an increased risk of coronary heart disease events and stroke with combined oestrogen-progestogen therapy, and no cardiovascular benefit with oestrogen alone, findings that fundamentally altered clinical practice and MHT prescribing patterns for nearly two decades [103,104].

Subsequent analyses and prospective trial evidence have substantially refined this picture through what is now termed the “timing hypothesis”. The ELITE trial (Early versus Late Intervention Trial with Estradiol) provided the most direct randomised evidence, demonstrating that oral oestradiol significantly reduced subclinical atherosclerosis progression in women who initiated therapy within six years of menopause, whereas no benefit was observed in those who initiated therapy more than ten years after menopause [105]. This temporal dependency implies that oestrogen may exert vasculoprotective effects on a relatively healthy, non-atherosclerotic arterial wall, whereas the same hormonal exposure may provoke plaque instability or thrombotic events in vessels already affected by established atherosclerosis.

The DOPS trial further supported this paradigm, reporting that MHT initiated at the time of menopause was associated with a significant reduction in the composite of death, myocardial infarction, and heart failure compared with no treatment over a ten-year randomised period. Importantly, no increase in cancer, venous thromboembolism, or stroke was observed in this early-initiation cohort, though the relatively small sample size limits definitive conclusions [106].

Current guidance from the European Society of Cardiology and the American Heart Association acknowledges that MHT is not indicated for primary or secondary prevention of cardiovascular disease, and that it carries an increased risk of venous thromboembolism, particularly with oral rather than transdermal formulations, as well as stroke at older ages [107]. However, MHT remains appropriate for the management of menopausal symptoms in women under the age of 60 or within ten years of menopause onset who are without established cardiovascular disease and should not be withheld solely on cardiovascular grounds in this population when symptom burden is significant [107]. For women with premature menopause, MHT is recommended until the natural age of menopause to mitigate the excess cardiovascular risk associated with prolonged oestrogen deficiency [107].

In clinical practice, the choice of MHT formulation, route of administration, and progestogen type carries cardiovascular relevance. Transdermal oestradiol appears to confer a lower thrombotic risk than oral preparations, and micronised progesterone may be preferable to synthetic progestogens from a metabolic and cardiovascular standpoint [108]. These distinctions, though not yet validated in adequately powered randomised outcome trials, inform individualised decision-making and warrant acknowledgment in the cardiovascular management of perimenopausal and postmenopausal women [109,110].

### 6.3. INOCA/ANOCA and CMD

INOCA is a highly related entity with women and requires therapeutic approach distinct from obstructive CAD. Among patients undergoing coronary angiography for angina, non-obstructive coronary arteries are reported far more frequently in women than in men, with estimates of approximately 50–70% in women compared with 30–50% in men.

The WISE study (Women’s Ischemia Syndrome Evaluation; n = 936 clinically stable symptomatic women) established the prognostic significance of INOCA long before the entity was formally recognized. Women with non-obstructive CAD had 10-year all-cause mortality and cardiac mortality rates of 17% and 11%, respectively, compared with 10% and 6% in women with entirely normal coronary arteries; approximately one in five enrolled women died from predominantly cardiac causes within 9 years of angiographic evaluation. A WISE sub study further demonstrated that impaired coronary flow reserve was an independent predictor of MACE over a median follow-up of 9.7 years (HR 1.06, 95% CI 1.01–1.12), quantifying microvascular dysfunction as a quantifiable prognostic marker [111].

The ISCHEMIA trial (International Study of Comparative Health Effectiveness with Medical and Invasive Approaches), which randomized 5179 patients with moderate-to-severe ischemia on stress testing to an invasive versus conservative management strategy, provided important sex-stratified data in a pre-specified analysis (1168 women [22.6%] and 4011 men [77.4%]). Women had substantially less extensive obstructive coronary disease than men: multivessel CAD was present in 60.0% of invasive-assigned women versus 74.8% of men, and no obstructive stenosis (≥50%) was identified in 12.3% of women versus only 4.5% of men (*p* < 0.001). Consequently, women underwent revascularization significantly less often than men in the invasive arm (73.4% vs. 81.2%; *p* < 0.001). Despite lower attainment of guideline-directed medical therapy goals, risk-adjusted primary outcome rates were similar between sexes (adjusted HR 0.93, 95% CI 0.77–1.13), with no significant sex-by-treatment-group interaction. Among patients excluded from ISCHEMIA due to INOCA, women had a fourfold higher odd of INOCA compared with men (OR 4.2, 95% CI 3.4–5.2), illustrating how inclusion criteria based on obstructive stenosis systematically exclude the phenotype most prevalent in women [112,113].

The role of invasive coronary function testing in guiding therapy was demonstrated in the CorMicA trial, a randomized, blinded clinical trial of patients with angina and no obstructive CAD [114]. Among 391 enrolled patients, 151 patients without the presence of obstructive disease in angiography were randomized to stratified medical therapy guided by an interventional diagnostic procedure or to standard care. Most randomized participants were women (73.5%). The diagnostic procedure identified isolated microvascular angina in 51.7%, isolated vasospastic angina in 16.6%, mixed microvascular and vasospastic angina in 20.5%, and non-cardiac chest pain in 11.3%. At 6 months, stratified therapy improved the Seattle Angina Questionnaire summary score by 11.7 units compared with standard care (95% CI: 5.0–18.4; *p* = 0.001), with parallel improvements in quality of life, while major adverse cardiac events did not differ between groups (2.6% vs. 2.6%; *p* = 1.00). These findings support the clinical value of endotype-guided diagnosis and treatment in patients with INOCA, a phenotype particularly relevant to women.

Treatment should be individualized according to the underlying mechanism. For vasospastic angina, calcium-channel blockers are generally first-line therapy, with long-acting nitrates added when symptoms persist. For microvascular angina with reduced coronary flow reserve or increased microvascular resistance, beta-blockers may be considered initially, followed by calcium-channel blockers if symptoms persist. Ranolazine, nicorandil where available, or enhanced external counter pulsation may be considered in refractory symptoms. ACE inhibitors or angiotensin receptor blockers and statins may be useful in women with endothelial dysfunction, impaired coronary flow reserve, hypertension, dyslipidemia, diabetes, or other cardiovascular risk factor [30].

The WARRIOR trial specifically enrolled symptomatic women with suspected INOCA [115]. In this randomized trial of 2476 women, intensive medical therapy with low-dose aspirin, high-intensity statin, and maximally tolerated ACE inhibitor or angiotensin receptor blocker did not significantly reduce the primary composite outcome compared with usual care at 5 years (17.84% vs. 16.17%). These neutral findings should not be interpreted as evidence against preventive therapy when conventional indications exist. Rather, WARRIOR highlights that a uniform treatment strategy may be insufficient for women with INOCA/ANOCA and reinforces the need for more precise, endotype-guided therapeutic trials.

### 6.4. MINOCA

MINOCA is particularly relevant in women and should be approached as a working diagnosis rather than a single disease entity. It accounts for approximately 5–10% of all myocardial infarctions overall, but its burden is disproportionately higher in women. In the ACTION Registry-GWTG, which included 322,523 patients with myocardial infarction, MINOCA was identified in 10.5% of women compared with 3.4% of men at angiography, highlighting an important sex-specific difference in acute coronary presentations [116] In a meta-analysis of nine studies including 30,281 MINOCA patients, of whom 18,079 were women and 12,202 were men, women had higher overall MACE rates than men over a median follow-up of 3.5 years (10.1% vs. 9.1%; OR 1.15, 95% CI 1.04–1.23). This excess risk was driven primarily by a higher incidence of stroke in women (3.5% vs. 2.2%; OR 1.30, 95% CI 1.01–1.68), whereas all-cause mortality did not differ significantly between sexes. These findings support careful follow-up and secondary prevention rather than reassurance after non-obstructive angiography [117].

Management should be mechanism-directed because MINOCA includes heterogeneous ischemic and non-ischemic mimics. In the PROMISE trial, patients randomized to a stratified strategy underwent comprehensive diagnostic assessment, including intracoronary imaging, vasomotor testing, cardiac magnetic resonance, and embolic-source evaluation when appropriate, followed by aetiology-guided therapy [118]. This approach identified the underlying mechanism in 80% of cases and reclassified the initial clinical diagnosis in 75.5% of patients. Compared with standard care, stratified treatment significantly improved angina-related health status at 12 months, with a Seattle Angina Questionnaire summary score difference of +9.38 points (95% CI 6.81–11.95; *p* < 0.001), while MACE was numerically lower but not statistically significant. Potential ischemic mechanisms include plaque disruption without obstructive stenosis, coronary vasospasm, coronary thromboembolism, coronary microvascular dysfunction, and SCAD. Cardiac magnetic resonance has emerged as a cornerstone of the structured MINOCA workup, particularly when the underlying cause is not identified, as it can help differentiate ischemic injury from myocarditis, Takotsubo syndrome, and other mimics while also providing prognostic information [119]. In a CMR-based cohort of 719 patients with ACS and non-obstructive coronary arteries, CMR established a diagnosis in 74% of cases; men more frequently had a non-ischaemic aetiology than women (55% vs. 41%; *p* < 0.001), whereas all-cause mortality did not differ significantly by sex over a median 4.9-year follow-up (8.7% vs. 10.1%; *p* = 0.456) [120,121]. These data reinforce the idea that women and men may present with different underlying mechanisms despite similar prognostic relevance, reinforcing the need for systematic multimodal evaluation rather than assuming that non-obstructive coronary arteries imply a benign or non-cardiac condition. Intravascular imaging with optical coherence tomography or intravascular ultrasound may be considered during index angiography to detect occult plaque rupture, plaque erosion, or SCAD, while provocative testing for coronary spasm may be considered in selected patients when no etiology is identified.

Therapy should be individualized according to the identified mechanism. Plaque-mediated MINOCA may warrant conventional secondary prevention, including antiplatelet therapy, statins, and risk-factors control. Vasospastic mechanisms should be treated with calcium-channel blockers and nitrates, while coronary microvascular dysfunction requires phenotype-guided antianginal therapy and aggressive risk-factors optimization. SCAD-related MINOCA should generally be managed conservatively when clinically stable, whereas Takotsubo syndrome should be separated from true ischemic MINOCA because its treatment is mainly supportive. Therefore, applying a uniform obstructive-CAD treatment strategy to all MINOCA patients is inappropriate.

The ongoing MINOCA-BAT trial (Randomized Evaluation of Beta-Blocker and ACE Inhibitor/ARB Treatment in MINOCA Patients, NCT03686696.) is prospectively evaluating the effect of beta-blockers and renin-angiotensin blockade on outcomes in this population, addressing a critical evidence gap where current secondary prevention strategies are extrapolated from obstructive CAD trials rather than derived from dedicated MINOCA data.

### 6.5. SCAD

SCAD a non-atherosclerotic cause of ACS most frequently suspected among younger, peripartum, or postpartum women presenting with ACS, especially when traditional atherosclerotic risk factors are absent or limited. In pregnancy-associated ACS, SCAD is a major mechanism and has been reported to account for approximately 43% of cases, most often during the third trimester or postpartum period [122,123].

In clinically stable women with preserved coronary flow and no ongoing ischemia, conservative management is generally preferred because spontaneous vessel healing is common, whereas PCI may extend the dissection or propagate the intramural hematoma. Revascularization should be reserved for high-risk presentations, including ongoing or recurrent ischemia, hemodynamic instability, left main coronary artery involvement, proximal vessel dissection supplying a large myocardial territory, or severely impaired coronary flow. This sex-specific distinction is clinically important because applying a routine obstructive-CAD revascularization strategy to SCAD may expose women to unnecessary procedural risk. Long-term management should include careful blood pressure control, individualized antiplatelet therapy, assessment for associated arteriopathies such as fibromuscular dysplasia when appropriate, and supervised cardiac rehabilitation, with treatment tailored to presentation, revascularization status, left ventricular function, and coexisting cardiovascular risk factors [122].

### 6.6. Takotsubo Syndrome

Takotsubo syndrome is another female-predominant ACS mimic and should be considered in women presenting with acute chest pain, electrocardiographic changes, and troponin elevation, particularly after emotional or physical stress. In the International Takotsubo Registry of 1750 patients, 89.8% were women, with a mean age of 66.8 years, supporting its strong association with postmenopausal female sex. Physical triggers were reported more often than emotional triggers, and a substantial proportion of patients had no identifiable trigger [124].

Initial evaluation may require coronary angiography to exclude obstructive ACS, because Takotsubo syndrome can closely mimic myocardial infarction. However, once the diagnosis is established and flow-limiting obstructive CAD has been excluded, PCI is not indicated because the syndrome is not caused by an epicardial culprit stenosis. Management is predominantly supportive and should be guided by clinical severity, left ventricular function, and complications.

Recognition is significant because postmenopausal women may otherwise undergo unnecessary invasive treatment or be misclassified as having plaque-mediated ACS. Clinical management should include monitoring for heart failure, arrhythmias, QT prolongation, electrolyte abnormalities, mitral regurgitation, ventricular thrombus, left ventricular outflow tract obstruction, and cardiogenic shock. In patients with cardiogenic shock, evaluation for left ventricular outflow tract obstruction is essential, as it occurs in approximately 20% of cases and directly influences hemodynamic management. Follow-up imaging should be performed to document recovery of ventricular function and guide the duration of heart-failure-directed therapy when used [125].

## 7. Transgender Women and Gender Diverse Individuals

Representation of women in clinical trials needs to be augmented based on the differences in phenotypes and pathophysiology, which can lead subsequently in personalization of care with improved clinical outcomes. Another aspect that needs to be altered is the inclusion of gender diverse individuals and transgender women. In accordance with the SAGER guidelines, sex and gender should be regarded as two discrete but interrelated constructs. Biological sex refers to chromosomal, anatomical, physiological traits, whereas gender encompasses socially and culturally influenced identities, roles and lived experiences that may affect health outcomes and access to healthcare [126].

Approximately 0.6% of the United States adult population identifies as transgender or gender diverse; however, these populations are rarely included in clinical trials [10,11,127]. According to the American Heart Association, data on cardiovascular risk factors for transgender and gender diverse individuals are limited, though there are several contributing factors that may worsen cardiovascular disease [127].

Minority stress—encompassing chronic exposure to misgendering, discrimination, and victimisation related to gender identity—has been associated with increased psychological stress, depression, anxiety, and adverse health behaviors, all of which may contribute indirectly to cardiovascular risk [128,129]. Due to psychological repercussions, higher rates of tobacco, alcohol, and substance use have been observed among transgender and gender-diverse individuals [130,131]. Hormone therapy may also affect cardiometabolic parameters. Based on epidemiologic studies, among transgender women, modest increases in triglycerides and reductions in HDL have been observed, varying according to the anti-androgen agent co-administered with oestradiol [132]. Moreover, higher triglycerides levels and lower HDL levels have been noted in transgender men on testosterone therapy while in some studies LDL may be elevated [133]. Long term cardiovascular implications of these findings remain incompletely understood.

Gender-affirming hormone therapy should therefore be considered in cardiovascular risk assessment, while also recognizing its established role in improving gender congruence and quality of life. Feminizing therapy commonly includes oestradiol with androgen suppression; cardiovascular risk may vary according to oestrogen formulation, route of administration, age, smoking status, and baseline thrombotic or cardiometabolic risk [134]. Transdermal oestradiol is generally considered preferable in individuals with higher thrombotic risk, while older ethinyl-oestradiol regimens are avoided because of their less favourable safety profile [135]. Masculinizing testosterone therapy may be associated with adverse lipid changes and erythrocytosis, requiring monitoring of lipids, blood pressure, glucose status, and haematocrit [136]. However, the long-term impact of gender-affirming hormone therapy on hard cardiovascular outcomes remains incompletely defined. Current expert guidance therefore supports individualized cardiovascular risk assessment, optimization of modifiable risk factors, and systematic collection of sex assigned at birth, current gender identity, hormone regimen, route, dose, and duration of exposure in clinical studies.

Nevertheless, data remains limited and it is of utmost importance to raise awareness and recruit transgender and gender diverse individuals in clinical trials, by collecting respective data such as, sex assigned at birth, current gender identity, hormone therapy exposure, duration of therapy, gonadal status where relevant, and stratified or sensitivity analyses when sample size allows, to better understand the cardiovascular risks to which are exposed.

## 8. Conclusions

In summary, while CAD remains the leading cause of death in both sexes, women continue to be underrepresented in cardiovascular research, and their distinct disease phenotypes still remain inadequately characterized. Cardiovascular disease in women more frequently manifests as MINOCA, SCAD, and microvascular dysfunction rather than obstructive epicardial atherosclerosis, and sex-specific risk enhancers including adverse pregnancy outcomes, premature menopause, PCOS, and autoimmune disease are insufficiently integrated into routine risk assessment and clinical trial design. Transgender and gender-diverse individuals represent a further and largely unaddressed evidence gap. Closing these disparities requires inclusive trial frameworks that capture sex, gender identity, and hormone exposure as distinct variables, alongside coordinated commitment from researchers, regulators, and funding agencies. Equity in representation in research should be the primary focus to advance equitable cardiovascular care in the future.

## Figures and Tables

**Table 1:** Clinical phenotypes of coronary artery disease and ischemic syndromes in women.

Phenotypes	Mechanism	Clinical Presentation	Key Diagnostic Modalities	Management Strategy
Obstructive CAD	Atherosclerotic plaque causing flow-limiting epicardial stenosis	Stable angina or ACS	Coronary computed tomography or invasive coronary angiography; physiological assessment with FFR/iFR when stenosis severity is uncertain	Guideline-directed secondary prevention, antianginal therapy, and revascularization when clinically indicated
Diffuse/non-calcified atherosclerosis	Widespread non focal plaque with discrete stenosis	Persistent ischemic symptoms with less focal obstructive disease	Coronary computed tomography or invasive coronary angiography; ± intracoronary imaging in selected cases.	Intensive risk-factor control, statin therapy, antiplatelet therapy when indicated, and symptom-directed antianginal treatment
Plaque erosion	Endothelial erosion with overlying thrombus without fibrous cap rupture	ACS, mostly in younger women, sometimes with limited angiographic obstruction	Intracoronary imaging (OCT preferred).	ACS-directed antithrombotic therapy, high-intensity statin therapy, and PCI only when clinically or anatomically required
INOCA/ANOCA/CMD	Endothelium dependent or independent microvascular dysfunction, impaired coronary flow reserve	Angina or ischemia despite non-obstructive coronary arteries	Reduced coronary flow reserve, abnormal microvascular resistance or abnormal stress perfusion imaging	Endotype-guided therapy, including risk-factor control, beta-blockers or calcium-channel blockers, ACEi/ARB, statins, nitrates or ranolazine as appropriate.
Vasospastic angina	Epicardial or microvascular smooth muscle hyperactivity	Recurrent rest angina, transient ischemic ECG changes, or symptoms with non-obstructive arteries	Epicardial or microvascular spasm on provocative testing in selected patients	Calcium-channel blockers as first-line therapy, long-acting nitrates or nicorandil when needed, and avoidance of triggers such as smoking or vasoconstrictive drugs
MINOCA	Plaque disruption, vasospasm, thromboembolism, microvascular dysfunction or SCAD	AMI with troponin rise but no obstructive coronary stenosis	Etiological workup with CMR, OCT/IVUS, and/or coronary function testing	Meechanism-based therapy: antiplatelet/statin therapy for plaque disruption, calcium-channel blockers for spasm, anticoagulation for embolic causes, and conservative SCAD management when appropriate
SCAD	Type 1: an angiographically visible intimal tear/double lumen, Type 2/3: Intramural hematoma causing coronary wall separation and luminal compression	ACS in younger, often peripartum or low-traditional risk women	Coronary angiography with careful interpretation; OCT/IVUS when angiography is inconclusive	Conservative management when stable, beta-blockers, aspirin when appropriate, PCI/CABG only for ongoing ischaemia, left main involvement, or haemodynamic instability
Takotsubo syndrome	Catecholamine-mediated adrenergic surge, causing temporary ventricular dysfunction	ACS like presentation often after emotional stress	Differentiate from ACS, myocarditis, and MINOCA using imaging and cardiac magnetic resonance	Supportive heart-failure therapy, treatment of complications, anticoagulation if LV thrombus or severe apical akinesia is present, and follow-up imaging to confirm recovery

## Data Availability

Not applicable.

## Abbreviations

ACE	Angiotensin-converting enzyme
ACS	Acute coronary syndrome
AMI	Acute myocardial infarction
ANOCA	Angina with non-obstructive coronary arteries
ARB	Angiotensin receptor blocker
CABG	Coronary artery bypass grafting
CAD	Coronary artery disease
CMR	Cardiac magnetic resonance imaging
CMD	Coronary microvascular dysfunction
CVD	Cardiovascular disease
DOPS	Danish Osteoporosis Prevention Study
EC	Endothelial cell
ECG	Electrocardiogram
ELITE	Early versus Late Intervention Trial with Estradiol
ESC	European Society of Cardiology
FMD	Flow-mediated dilation
HDL	High-density lipoprotein
HR	Hazard ratio
IHD	Ischemic heart disease
INOCA	Ischemia with non-obstructive coronary arteries
IVUS	Intravascular ultrasound
LDL	Low-density lipoprotein
LV	Left ventricle
LVOT	Left ventricular outflow tract
MACE	Major adverse cardiovascular events
MHT	Menopausal hormone therapy
MINOCA	Myocardial infarction with non-obstructive coronary arteries
MRI	Magnetic resonance imaging
NID	Nitroglycerin-induced dilation
NO	Nitric oxide
OCT	Optical coherence tomography
OR	Odds ratio
PCI	Percutaneous coronary intervention
PCOS	Polycystic ovary syndrome
PMOS	Polyendocrine metabolic ovarian syndrome
RAS	Renin-angiotensin system
ROS	Reactive oxygen species
RR	Risk ratio
SAGER	Sex and Gender Equity in Research
SCAD	Spontaneous coronary artery dissection
STEMI	ST-elevation myocardial infarction
SWAN	Study of Women’s Health Across the Nation
WHI	Women’s Health Initiative
WISE	Women’s Ischemia Syndrome Evaluation
